# A comparative study to identify factors of caregiver burden between baby boomers and post baby boomers: a secondary analysis of a US online caregiver survey

**DOI:** 10.1186/s12889-018-5488-4

**Published:** 2018-05-02

**Authors:** Heejung Kim, Sangeun Lee, Jooyoung Cheon, Soyun Hong, Mido Chang

**Affiliations:** 1 College of Nursing, Yonsei University, 50-1 Yonsei-ro, Seodaemun-gu, Seoul, 03722 Republic of Korea; 2 Mo-Im Kim Nursing Research Institute, Yonsei University, 50-1 Yonsei-ro, Seodaemun-gu, Seoul, 03722 Republic of Korea; 3 College of Nursing, Sungshin Women’s University, 55 Dobong-ro 76ga-gil, Gangbuk-gu, Seoul, 01133 South Korea; 4 Department of Leadership & Professional Studies, School of Education & Human Development, College of Arts, Sciences & Education, Florida International University, 11200 SW 8th St. ZEB 250B, Miami, FL 33199 USA

**Keywords:** Caregiver burden, Age, Baby boomers, Post baby boomers, National Alliance for Caregiving, Secondary data analysis

## Abstract

**Background:**

Baby boomers’ position in the caregiving context is shifting from caregiver to care recipient as the population ages. While the unique characteristics of baby boomer caregivers are well established in caregiving literature, there is limited information about the next caregiving group after the baby boomers. In this study, the sociodemographic and caregiving-related characteristics of the two generations are compared and specific factors contributing to caregiver burden between baby boomer and post baby boomer caregivers are identified.

**Methods:**

This cross-sectional and correlational study used secondary analysis of data from the National Alliance for Caregiving and the American Association of Retired Persons. A structured online survey was conducted in 2014 with randomly selected samples (*n* = 1069) in the United States focusing on sociodemographics, caregiving-related characteristics, and burden of care. Descriptive statistics, multivariate linear regression analyses, and Steiger’s Z-test were used to identify group differences in multivariate factors related to caregiver burden in two generational groups.

**Results:**

Baby boomers and post baby boomers experienced caregiver burden to a similar degree. Caregiving-related factors are more likely to increase burden of care than sociodemographics in both groups. Caregiving without choice and spending longer hours on caregiving tasks were common factors that increased the burden in both generational groups (all *p* values < 0.01). However, post baby boomer caregivers reported additional challenges, such as unemployment during caregiving, the dual responsibility of both adult and child care, and a family relationship with the care recipient.

**Conclusions:**

Due to the aging population of baby boomers, post baby boomers encounter different challenges related to caregiving burden, which is often considered an additional workload in their life course. Current policy and program tailored to baby boomers should be re-designed to meet the different needs of emerging caregivers. Specific vulnerable subgroups should have priority to receive the benefits of specific policies, such as those without choice and younger, working caregivers.

## Background

Informal caregiving is more important than ever because of an aging population, increasing healthcare expenditures, and the lifelong impact of chronic diseases. In 2013, seven developed countries established the International Alliance of Carer Organizations to advocate for caregivers’ health and to acknowledge their values. The priority action in their strategic plan is to increase awareness of caregiving, with a focus on who caregivers are and what they do worldwide [1]. As a unique cohort due to the cultural shift and historical background [2, 3], baby boomer caregivers provide more and longer caregiving for aging parents than ever before as life expectancies increase. In addition, they continue to support their grown children financially, even in adulthood [4–9]. However, baby boomers’ position in the caregiving context is shifting from caregiver to care recipient [3, 7] because they are aging as a population [10], with high demands on their own health [4, 5]. In 2015, average ages of informal caregivers and care recipients in the U.S. were 49 and 69 years old, respectively, which indicates that the next generation after baby boomers are expected to play a critical role in informal caregiving.

Caregiving provided by post baby boomers is expected to be different from that of previous generations because of change of culture and family systems. Post baby boomer caregivers were born into smaller families because of the introduction of contraceptives and grew up in a time when divorce rates were high [11, 12]. Their mothers worked outside the home more compared to the mothers of earlier generations, which led to them being called “latchkey children” [11, 12]. Parent-child relationships among post baby boomers are weaker than parent-child relationship among baby boomers because of parents’ divorces, remarriages, and multi-partnerships [12, 13].

Although the unique characteristics of baby boomer caregivers are well established in terms of their health problems, financial hardships, and role perceptions [5, 6, 14], healthcare providers and policy makers have paid less attention to upcoming caregivers who will care for baby boomers. Baby boomers might expect high levels of informal caregiving because they have provided their offspring strong support [9, 15]. However, there is limited information on the next caregiving group after the baby boomer generation [9, 11, 14]. Only two studies have examined generation differences between baby boomers and post baby boomers; however, they examined generational differences in self-rated health [16] and work-family conflict [17].

In addition, caregivers need to perform diverse roles across the life span in terms of family-work balance [18]. Different factors of increasing burden of care seem to be associated with levels of family involvement, caregiving duties, and different challenges caregivers encounter at specific points in their lifespan [19–21]. Younger caregivers want to be more active when participating in social activities, enjoying leisure, or performing working tasks compared to older caregivers, while older caregivers feel responsible for prioritizing the caregiving role rather than their social roles [20, 22]. However, it is questionable whether this trend will continue, because of baby boomers’ unique characteristics in pursuing independence, autonomy, and strong healthcare needs [4, 5]. Moreover, most previous studies examined informal caregivers of persons living with specific diseases, such as dementia [23] and cancer [24], thus it is difficult to apply study findings to the overall caregiving population [23–25]. Therefore, the objectives of this study are to examine the sociodemographic and caregiving-related differences between baby boomer caregivers and post baby boomer caregivers and to compare the contributing factors to caregiver burden between two generations.

## Methods

### Design

This cross-sectional, correlational study incorporated secondary data analysis of the National Alliance for Caregiving (NAC) and the American Association of Retired Persons (AARP) [2] database.

### Samples and procedure of secondary data analysis

**Fig. 1:**
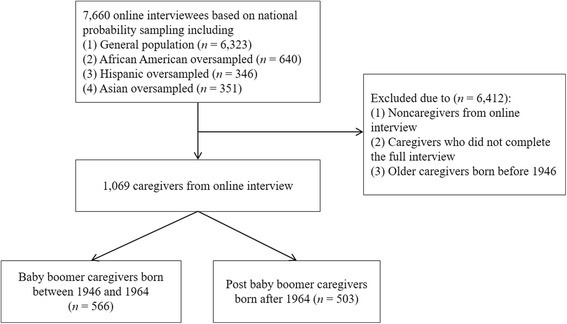
Flowchart of samples describes how to reach the final sample (*n* = 1069) samples (unweighted) from 7660 online interviewees

For the secondary data analysis, an exempt status was obtained through the Institutional Review Board based on the use of de-identifiable data. Informal caregivers were defined as persons providing unpaid care or assistance based on needs or performing housework for those whom the respondent knew [4]. To compare two generational groups of caregivers, we categorized baby boomers born between 1946 and 1964 and post baby boomers born after 1964 using the U.S. nationally representative samples. In the primary data of the NAC and AARP survey, a nationally representative sample in the U.S. (*N* = 7660) was selected based on (1) a random selection of telephone numbers and residential addresses and (2) oversampling of racial and ethnic minority groups [26]. The analysis included 1069 persons after excluding those who did not meet eligibility criteria (see Fig. 1).

**Fig. 2:**
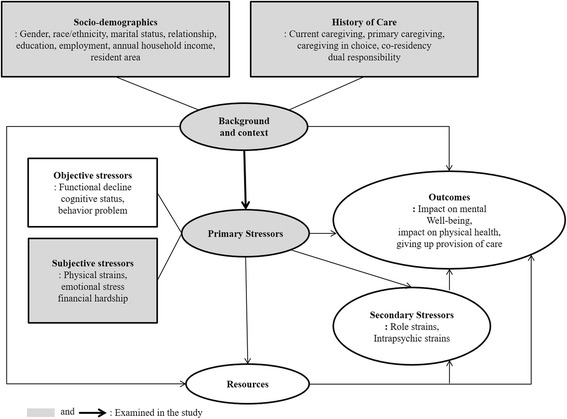
Conceptual framework of Pearline’s stress process model shows a modified model that guided variable selection and interpretation of the study findings

For primary data collection in the NAC and AARP survey, a standardized online questionnaire was administered via a computer-aided Web interviewing system, in English or Spanish, in September 2014 after obtaining informed consent [26]. The variables for this secondary data analysis were selected from the 2015 NAC and AARP data according to Pearlin’s Stress Process model (see Fig. 2) [27]. First, caregivers’ sociodemographics included age, gender, race/ethnicity, marital status, a relationship to the care recipient, education, co-resident status with care recipients, annual household income, and residence area. Household income was dichotomized based on U.S. $50,000 per year, which is considered the median income level [28]. Second, information on caregiving included (1) caregiving hours spent on care recipients per week, (2) current caregiving during the past 12 months, (3) primary caregiving with or without secondary caregivers, (4) lack of choice to take the caregiving role, (5) employment during caregiving, and (6) dual responsibility for both adult and child care. Third, self-reported caregiver burden consisted of three items—physical strain, emotional stress, and financial hardship—as the previous studies used to assess the health of caregivers more comprehensively [14, 15]. Each item was measured on a 5-point Likert scale (1 = not at all a strain/stressful/hardship; 5 = very much a strain/stressful/hardship). A composite score was computed with the mean of the three items, consistent with the NAC and AARP report and a previous study [29] (Cronbach’s alpha = 0.73).

### Analysis

Descriptive statistics included means with standard deviations and weighted percentages. Independent t-tests (or Mann Whitney U-tests) and *χ*^2^-tests were used to compare sociodemographic and caregiving characteristics between two groups. Multivariate linear regression and Fisher’s and Steiger’s Z-tests [30] were used to identify and compare significant predictors of caregiver burden between the two groups. The Z-test is computed as (Z_1_-Z_2_)/SE_ZD_ and SE_ZD_ = squared root of [1 / (n1–3) + 1 / (n2–3)]. The number of hours of caregiving per week was transformed using a natural log function considering univariate normality. There was no concern regarding (1) linearity between caregiving burden and the transformed variable of the number of hours of caregiving per week and (2) multicollinearity among independent variables. All statistical analyses were performed using IBM SPSS 23.0 with the significance level set at .05, two-tailed. A single-stage population weighting was used to increase generalizability of the study findings to national caregivers at the population level. The population weighting was based on information of initially screened and randomly selected respondents. Each score was calculated based on age, sex, and race/ethnicity information extracted from the 2014 Current Population Survey conducted by the U.S. Census Bureau [26].

## Results

### Sociodemographic differences between baby boomer and post baby boomer caregivers

The average age of the caregivers was 42.25 (*SD* = 13.78) and ranged from 18 to 68. The average age in the baby boomer group was 57.90 (*SD* = 5.28), while that in the post baby boomer group was 34.75 (*SD* = 9.00). The majority of caregivers were female, non-Hispanic Caucasian, married or partnered, had more than a high school education, were employed, and lived in urban areas. Half of the caregivers had an annual household income of more than $50,000 and were the care recipient’s child or grandchild (see Table 1). Compared to post baby boomer caregivers, baby boomer caregivers were likely to be non-Hispanic Caucasian, married or living with a partner, and living with more household income than $50,000 per year (*p* ≤ 0.01). Employment during caregiving was lower in the baby boomer group than in the post baby boomer group (*χ*^*2*^ = 11.34, *p* < 0.01). Baby boomer and post baby boomer caregivers were the care recipients’ child or grandchild; however, baby boomers were more likely to be a spouse or parent of the care recipient (*χ*^*2*^ = 37.20, *p* < 0.01). There were no significant differences in gender, education, and residential area between the two generational groups.

**Table 1:** Comparison of sociodemographic characteristics of informal caregivers in the two generational groups

Variables	Levels	All (weighted %)	Baby boomer group (weighted %)	Post baby boomer group (weighted %)	*χ* ^*2*^	*p* value
Gender	Male	40.94	38.45	43.01	2.24	0.15
	Female	59.06	61.55	56.99		
Race/ethnicity	NH Caucasian	59.36	71.10	49.65	51.19	< 0.001
	NH African American	13.48	9.49	16.78		
	Hispanic	18.36	12.03	23.60		
	NH Asian or others	8.80	7.38	9.97		
Marital status	Married or partnered	65.01	69.51	61.25	7.66	0.01
	No partner	34.99	30.49	38.75		
Relation to the care-recipient	Spouse or partner	8.03	10.55	5.95	37.20	< 0.01
	Parents or grandparents	4.11	7.59	1.22		
	Child or grandchild	54.49	50.21	58.04		
	Other relative	19.41	19.41	19.41		
	Non-relative	13.96	12.24	15.38		
Education	Up to high school	35.11	34.74	35.43	0.05	0.85
	College level or higher	64.89	65.26	64.57		
Co-residence	Live with care-recipients	32.75	31.06	34.16	1.12	0.32
	Live separately	67.25	68.94	65.84		
Household poverty	Less than $50,000	46.70	40.21	52.10	14.73	< 0.01
	More than $50,000	53.30	59.79	47.90		
Residence area	Urban	84.73	83.16	86.04	1.67	0.23
	Rural	15.27	16.84	13.96		

*NH* Non-Hispanic

### Caregiving-related differences in two caregiving groups

Caregivers spent an average of 24.42 (*SD* = 29.18) hours weekly on caregiving. The baby boomer group spent 4 h more on caregiving (M = 25.76, SD = 29.43) than the post baby boomer group (M = 21.73, SD = 27.69, *p* < 0.01). Baby boomers reported higher levels of caregiver burden (*M* = 2.64, *SD* = 1.03) compared to post baby boomers (*M* = 2.56, *SD* = 1.04, *p* < 0.04). Both generational groups were currently providing caregiving, living separately from the care recipient, and taking care of adult care recipients only; however, these tendencies were stronger in baby boomer caregivers than in post baby boomer caregivers. Post baby boomers were likely to be primary caregivers rather than secondary caregivers, but they chose to be caregivers based on their own decision. Caring for both adult and child recipients was significantly higher in the post baby boomers (22.90%) compared to the baby boomer group (6.95%, *χ*^*2*^ = 50.00, *p* < 0.001, see Table 2).

**Table 2:** Caregiving-related characteristics of informal caregivers between the two generational groups

Variables	Levels	All (weighted %)	Baby boomer group (weighted %)	Post baby boomer group (weighted %)	χ^2^	*p* value
Current caregiving	Currently providing	54.87	58.74	51.66	5.26	0.02
	Provided in the past 12 months	45.13	41.26	48.34		
Primary caregiving	Primary provider	61.10	59.07	62.79	1.50	0.23
	Non-primary provider	38.90	40.93	37.21		
Caregiving lack of choice	Lack of choice	50.00	55.79	45.17	11.68	< 0.01
	Caregiving by choice	50.00	44.21	54.83		
Employed during caregiving	Employed during caregiving	68.13	62.82	72.55	11.34	< 0.01
	Unemployed during caregiving	31.87	37.18	27.45		
Dual responsibility	Adult care only	84.34	93.05	77.10	50.00	< 0.001
	Both adult and child care	15.66	6.95	22.90		

### Model comparison of caregiver burden between two generational groups

As shown in Table 3, the model of caregiver burden for baby boomers significantly explained 18% of the variance, which was lower than 21% in the model for the post baby boomers (*p* < 0.001) after controlling for age. In both generational groups, caregiving-related characteristics (Block 2) explained 2.5 times as much of the variance in caregiver burden as sociodemographic characteristics (Block 1). A comparison of the two generational groups was conducted by applying the model derived from the baby boomer group (direct *R*^*2*^ = 0.44) to the data from the post baby boomer group (crossed *R*^*2*^ = 0.44). However, two models were not statistically different (*Z* = 1.16, *p* = 0.25).

**Table 3:** Results of multiple regression analyses

			Baby boomer group			Post baby boomer group			Comparison of regression coefficients	
Blocks	Variables		B	SE	beta	B	SE	beta	SE_b-diff_	Z
Block 1. Sociodemographic factors	Gender	Female	−0.08	0.09	−0.04	−0.03	0.08	−0.01	0.12	−0.42
	Race-ethnicity	NH African-Americans	−0.02	0.18	0.00	0.07	0.12	0.02	0.22	−0.42
		Hispanics	−0.31*	0.15	−0.09	− 0.13	0.10	− 0.05	0.18	−1.00
		NH Asian and others	0.12	0.19	0.03	−0.06	0.16	−0.02	0.25	0.72
	Marital status	Not married or partnered	0.03	0.11	0.01	−0.14	0.09	−0.07	0.14	1.20
	Relationship to the care recipient	Spouse	0.31	0.23	0.09	0.47*	0.22	0.11	0.32	−0.50
		Child	0.28	0.15	0.14	0.40**	0.13	0.19	0.20	−0.60
		Parent	0.41	0.21	0.11	0.11	0.38	0.01	0.43	0.69
		Other relative	0.09	0.17	0.04	0.32*	0.15	0.12	0.23	−1.01
	Co-residence	Live separately	0.10	0.12	0.05	0.11	0.10	0.05	0.16	−0.06
	Household poverty	Less than $50,000 yearly	0.05	0.10	0.02	0.08	0.09	0.04	0.13	−0.22
Block 2. Caregiving-related factors	Caregiving hours spent weekly		0.17***	0.03	0.26	0.10***	0.03	0.16	0.04	1.65
	Current caregiving		−0.02	0.09	−0.01	− 0.08	0.08	− 0.04	0.12	0.50
	Primary caregiving		−0.11	0.10	−0.05	0.08	0.09	0.04	0.13	−1.41
	Caregiving lack of choice		0.59***	0.09	0.29	0.65***	0.09	0.31	0.13	−0.47
	Employed during caregiving		0.11	0.09	0.05	−0.19*	0.09	−0.08	0.13	2.36*
	Dually responsible for adult and child care		0.24	0.18	0.06	0.27**	0.10	0.11	0.21	−0.15
Adjusted *R*^*2*^	Δ*R*^*2*^ for Block 1		0.05***			0.06***			−0.27	
	Δ*R*^*2*^ for Block 2		0.13***			0.15***			−0.53	
	Total		0.18***			0.21***			1.16	

*SE* Standard Error, *B* unstandardized regression coefficient, *SE*_*b-diff*_ difference in standard error of unstandardized regression coefficient, *Z* Steiger’s Z, *NH* Non-Hispanic, Δ*R*^*2*^ change of R-squared

*p < 0.05, **p < 0.01, ***p < 0.001

After controlling for caregivers’ socio-demographic characteristics including age, caregiving without choice was identified as the most significant factor between the two generational groups (*β =* 0.29 in baby boomer group and *β =* 0.31 in the post baby boomer group, *p* < 0.01) to similar degrees (*Z* = − 0.47, *p* = 0.32). The number of hours of caregiving was another common factor contributing to caregiver burden in both generational groups (*β =* 0.26 in the baby boomer group and *β =* 0.16 in the post baby boomer group, *p* < 0.01) to similar degrees (*Z* = 1.65, *p* = 0.05). Employment during caregiving showed a statistically significant difference (*Z* = 1.65, *p* = 0.05): the post baby boomers reported lower levels of burden when they were employed during caregiving; however, baby boomers reported a higher burden during employment.

In addition, few differences in individual factors were reported. First, Hispanic baby boomers reported lower levels of caregiver burden than non-Hispanic Caucasians (*β =* − 0.09, *p* = 0.04). Second, relationships between caregivers and care recipients were identified as a significant factor of caregiver burden in the post baby boomer group, but not for baby boomers. Compared to nonfamily caregivers, family caregivers in the post baby boomer group reported higher levels of burden when the care recipient was a spouse, child, or other relative (respectively, *β =* 0.11, 0.19, and 0.12; all *p* values < 0.01). In addition, the post baby boomer group reported higher levels of burden when they took care of both adults and children simultaneously compared to those responsible for adult care recipients only (*β =* 0.11*, p* < 0.01).

## Discussion

In this study, caregiving-related factors were more likely to increase burden than sociodemographics, specifically, caregiving without choice and longer hours spent on caregiving in both generational groups. However, post baby boomers reported additional challenges during caregiving, such as dual responsibility of both adult and child care, unemployment, and a family relationship with the care recipient.

Caregiving by choice was the most important factor that determined the level of caregiver burden. Taking a caregiving role without choice is considered stressful regardless of the presence of secondary assistants or a co-resident situation [10, 31]. It is important for caregivers to have the opportunity to voluntarily take on the role after they have considered the benefits in relation to the autonomy and self-determination they would give up by becoming a caregiver [31]. After careful consideration, undecided or potential candidates would take on the caregiving role after evaluating the expected gains and losses to their own future plans [10, 32].

Interestingly, our study findings show that the post baby boomers spent significantly less time on caregiving compared to baby boomers. Usually, a greater number of caregiving hours means a more severe medical condition or functional decline in the care recipient, which is considered an increasing factor of caregiving burden [29, 31]. The use of formal health services among older baby boomers might also influence the time spent on caregiving in the post baby boomer group [33]. However, another study has found no relationship between the number of hours and stress level during caregiving [14]. Further research should use objective methods to measure the actual time spent, rather than relying on self-reporting by caregivers, to examine the unique effect of time spent on each care recipient.

Higher burden of care associated with dual caregiving responsibility was identified in the post baby boomer group. Recently, baby boomers have been moving into the aging population and out of the sandwich generation [7]. Younger caregivers with dual responsibilities are at risk of feeling pressed for time and stressed because of the time management requirements of daily life [7]. However, in our study, the tendency to spend less time on caregiving reflected their time management strategies, in contrast to a previous study, in which caregivers with dual responsibilities spent more time on caregiving [8]. They might be proactive in requesting assistance from secondary caregivers, which decreases time spent on adult care [34]. When considering the higher proportion of dually responsible caregivers in the post baby boomer group, younger caregivers might subtract the time spent on child care from the total time of caregiving because raising children is an expected role in their developmental stage [8, 10]. Further studies should perform moderation analyses of caregiver burden in those with dual responsibilities, by generational group or resource use, as in a previous study [8].

Unemployment was a unique factor contributing to caregiver burden in the post baby boomer group. The annual income of the post baby boomer caregivers was significantly lower than the baby boomer caregivers, although the majority of both generational groups were likely to be working during caregiving [35]. A previous study reported that the strength of negative impact on caregiving burden varies depending on levels of income [15]. Loss of regular income has been identified as a significant factor in decreasing financial security, livelihood, self-worth, and social interaction [36, 37]. Thus, financial hardships may make the younger group feel vulnerable as part of their caregiver burden.

Post baby boomer caregivers reported a high burden when caring for any family member or relative except their parents. Historically, family members take on caregiver roles as a normative expectation and learn tasks without purposive effort [5]. The predictable demands of child care were relatively acceptable and prepared them for performing specific tasks and roles. However, caring for young spouses and other relatives with diverse healthcare needs was less predictable and more likely to worsen over time across the caregiving trajectory [31, 38]. Many post baby boomer caregivers are raised by a single parent. They may maintain family ties with their parents, but not with other family members or relatives because of changes in marital disruptions, weak family ties, and romantic patterns [12, 13].

As the baby boomers have joined the aging population, it is necessary to revisit the current social policy programs that were mostly designed for baby boomer or older caregivers [39, 40]. In addition, awareness programs should include the importance of voluntary decisions in taking caregiving roles and the ability to balance between gain and loss during caregiving [31, 32]. For vulnerable caregivers, including those who had no choice in taking on their caregiving role and higher-hour caregivers, support to hire paid helpers, access to paid family leave benefits through their employers, cash compensation for their unpaid caregiving hours, and provision of an income tax credit for loss of work hours due to caregiving would be most helpful [2]. A flexible work schedule and employer-sponsored day-care centers for both adult and child care recipients are also helpful for working caregivers [6].

Our study has several limitations. First, the use of cross-sectional data limited the study’s ability to make predictive statements about the factors contributing to caregiver burden. Second, we used self-report data obtained from caregivers without information on care recipients. Third, age may affect all other independent variables because the two age groups (baby boomers and post baby boomers) occupy different stages of the life cycle. Future studies could consider dyadic relationships between caregivers and care recipients by adding information about the medical conditions of the care recipients. Longitudinal studies need to include validated multiple measures of psychological distress for comprehensive understanding of caregiver burden change over the lifetime within a specific age group.

## Conclusion

This study examined sociodemographic and caregiving-related characteristics of informal caregivers in the United States and specified factors associated with subjective burden of care in each generational group. By comparing two generational caregiver groups, baby boomers and post baby boomers experience unique age-related challenges associated with burden of care were identified. Compared to baby boomer group, post baby boomer caregivers are experiencing more time constraint and financial problems that conflict with their other responsibilities at home and work. Our study suggests that it is necessary to develop age-specific caregiving programs and resources tailored to the developmental needs of informal caregivers in different stages of life. In addition, specific vulnerable groups should have priority to receive the benefits of policies, such as those without choice and younger working caregivers.

## Abbreviations

AARP: The American Association of Retired Persons
NAC: National Alliance for Caregiving
NH: Non-Hispanic
